# Ecological insights into the cross-domain microbiome interactions in the hematophagous bat *Desmodus rotundus*

**DOI:** 10.1186/s42523-025-00504-x

**Published:** 2026-02-19

**Authors:** Nicolas Luna, Carolina Hernández, Angie L. Ramírez, Plutarco Urbano, Karen Barragán, Catalina Ariza, Marina Muñoz, Luz H. Patiño, Juan David Ramírez

**Affiliations:** 1 Centro de Investigaciones en Microbiología y Biotecnología - UR (CIMBIUR), School of Sciences and Engineering, Universidad del Rosario, Bogotá, Colombia; 2 Center for Global Health and Interdisciplinary Research, USF Genomics Program, Department of Global, Environmental and Genomic Health Sciences, College of Public Health, University of South Florida, Tampa, FL USA; 3 Centro de Tecnología en Salud (CETESA), Innovaseq SAS, Bogotá, Colombia; 4 Grupo de Investigaciones Biológicas de la Orinoquia, Universidad Internacional del Trópico Americano (Unitrópico), Yopal, Colombia; 5 Instituto de Biotecnología-UN (IBUN), Universidad Nacional de Colombia, Bogotá, Colombia

**Keywords:** Microbial communities, Common vampire bat, Long-read sequencing, Feeding sources, Microbial diversity and community structure, Zoonotic pathogens

## Abstract

**Background:**

Bats are recognised as reservoirs for a wide range of microorganisms, including viruses, bacteria, fungi, and parasites, some of which are of zoonotic concern. The common vampire bat (*Desmodus rotundus*) is particularly important due to its hematophagous feeding behaviour and ecological adaptability, both of which enhance its potential for cross-species pathogen transmission. Despite its well-established relevance to public health, the microbial communities associated with *D. rotundus* remain poorly characterised. This study aimed at investigating the composition, diversity, and interactions of prokaryotic, eukaryotic, and viral communities, alongside feeding sources, using high-throughput sequencing in 27 *D. rotundus* individuals from a rural area in Casanare, eastern Colombia.

**Results:**

We analysed a total of 81 samples (blood, faeces, and oral swabs) using long-read amplicon sequencing of the 16S- and 18S-rRNA genes and viral metagenomics via Oxford Nanopore Technologies. The microbial profiles revealed highly diverse assemblages, encompassing a wide range of bacterial, fungal, eukaryotic parasites, and viral taxa, with significant variation in community structure and diversity metrics across the three sample types collected from each bat. Taxa of public health concern were detected, including *Enterococcus faecalis*, *Mycoplasma* spp. *Acanthamoeba* spp. and viruses from the families *Coronaviridae*, *Retroviridae*, and *Circoviridae*. Correlation analyses suggested potential intra- and inter-domain interactions and co-occurrence dynamics among these microbes. Additionally, feeding source profiling, based on vertebrate assignments from faeces and swab samples, indicated evidence of livestock consumption, suggesting possible transmission pathways between bats and domestic animals.

**Conclusions:**

The detection of multiple co-occurring pathogens across distinct sample types, coupled with their association with feeding sources, highlights the role of *D. rotundus* as a functionally specialised reservoir capable of harbouring and potentially disseminating zoonotic microbes. This study provides new insights into the cross-domain microbial ecology of hematophagous bats and underscores the need to integrate microbial community profiling with host behavioural data to enhance surveillance and mitigation strategies for zoonotic disease transmission.

**Supplementary information:**

The online version contains supplementary material available at 10.1186/s42523-025-00504-x.

## Background

Bats (Chiroptera) are a diverse group of mammals that play multiple functional roles in the ecosystems they inhabit, acting as pollinators, seed dispersers, and keystone species in regulating populations of insects and small mammals [1–3]. In addition to their ecological importance and broad geographic distribution, inhabiting a variety of ecosystems (rural, urban and forest) [4, 5], these mammals exhibit diverse feeding habits (frugivorous, insectivorous, piscivorous, carnivorous, nectivorous, omnivorous and hematophagous), enabling them to colonize and exploit different environments [6, 7]. Furthermore, bats are of public health relevance, serving as hosts for ectoparasitic vectors (e.g., ticks, fleas, and mites) [8], and notably, as hosts or reservoirs of various pathogenic microorganisms, including viruses, bacteria, parasites and fungi [9, 10]. Their capacity to tolerate, harbour, and transmit a wide range of pathogens may be related to their unique ability to fly [11, 12], which has driven the evolution of distinct genomic and immunological traits that allow them to maintain both physiological homeostasis and microbial activity [12–14].

Among bat species, the common vampire bat (*Desmodus rotundus*) stands out as one of the three species of hematophagous bats. This species is widely distributed throughout tropical and subtropical regions of the Americas, inhabiting diverse ecosystems, including forested, rural, and urban areas [15]. Ecologically, *D. rotundus* exhibits a complex social structure [16] and ecological plasticity, enabling it to colonise a variety of environments [17]. Unlike the other hematophagous species, the hairy-legged vampire bat (*Diphylla ecaudata*) and the white-winged vampire bat (*Diaemus youngii*), *D. rotundus* feeds on the blood of a wide range of vertebrates, mainly mammals [17–19]. Due to its feeding strategy, this species has been proposed as a key indicator of vertebrate diversity in wild ecosystems [20]. Nonetheless, anthropogenic pressures such as livestock intensification, deforestation, and urban expansion have altered its distribution and ecological dynamics. These environmental changes have not only displaced *D. rotundus* into new habitats but have also shifted its feeding behaviour [17, 21]. As a result, the expansion of *D. rotundus* into livestock-dense areas has caused significant economic losses [22]. This is mainly due to a long-term increase in blood-feeding on cattle and other domestic animals, which has led to reduced productivity, higher veterinary costs, and livestock mortality [21, 23]. In addition to its economic impact, *D. rotundus* is of public health concern due to its association with various infectious diseases.

From a public health perspective, *D. rotundus* is known as a natural reservoir for several pathogenic microbes, including bacteria (*Brucella* and *Bartonella*) [24, 25], parasites (*Trypanosoma cruzi* and Piroplasmids) [26–28], and viruses (*Rhabdovirus*, *Coronavirus*, *Adenovirus*, and *Herpesvirus*) [29–32]. Notably, its hematophagous feeding habits further elevate the risk of spillover events, where pathogens are transmitted from bats to other species [33]. These bats are considered the principal natural reservoir of the rabies virus (RABV), transmitting it through bites to different mammals, especially domestic animals and livestock [34]. Due to their relevance to both human and animal health, several Latin American countries have implemented pathogen-focused genomic surveillance programmes to monitor and prevent the emergence and spread of zoonotic diseases associated with *D. rotundus*, mainly viral diseases such as RABV [35, 36]. To date, multiple pathogens have been identified in these bats, including *Trypanosoma cruzi*, *Bartonella*, and different viruses [25, 28], highlighting their role in the transmission cycles and routes of multiple infectious agents [17].

Using next-generation sequencing tools, several studies have identified and described multiple emerging and re-emerging pathogens in bats. These techniques have enabled the molecular characterization of diverse microorganisms, as well as the analysis of their genomic, ecological, and evolutionary features [37–39]. In the common vampire bat, infectious agents have been detected using specific molecular markers, including the ribC gene for *Bartonella*, the L gene for rhabdoviruses, the polymerase gene for herpesviruses [40], and the 18S rRNA gene for piroplasmids and *Trypanosoma* [27, 28]. Likewise, metagenomic approaches have facilitated the identification of a wide variety of viral communities of relevance and the discovery of novel microorganisms [35, 41, 42]. In addition to characterising microbes, these techniques also provide an understanding of the vertebrate species that comprise the diet of hematophagous bats [43], offering insights into feeding patterns and potential routes of disease transmission. However, despite the characterisation of specific pathogens, few studies have analysed the ecology of the different microbial communities of these mammals.

In bats, microbial communities comprise different microorganisms, many of which are pathogenic to other organisms [9, 10], with functional roles linked to the ecological characteristics of their hosts [44, 45]. Ecologically, the diversity and abundance of these communities may be determined by geography, social interactions, the ecological traits of the bat and even its physiological features [46–48]. These determinants shape communities associated with particular bat species [45] or specific to a physiological system [47, 49]. Although several studies in *D. rotundus* have described a functional microbiome adapted to its hematophagous habits [13, 46], most have focused on bacteria and viruses. Consequently, little is known about the ecological characteristics of other microbial communities, such as fungi and parasites, which also play an important role in the transmission of infectious diseases in these bats [9]. Furthermore, the complete picture of microbial communities (bacteria, fungi, parasites and viruses) and their ecological relationships with hematophagous bats are also unknown.

**Fig. 1:**
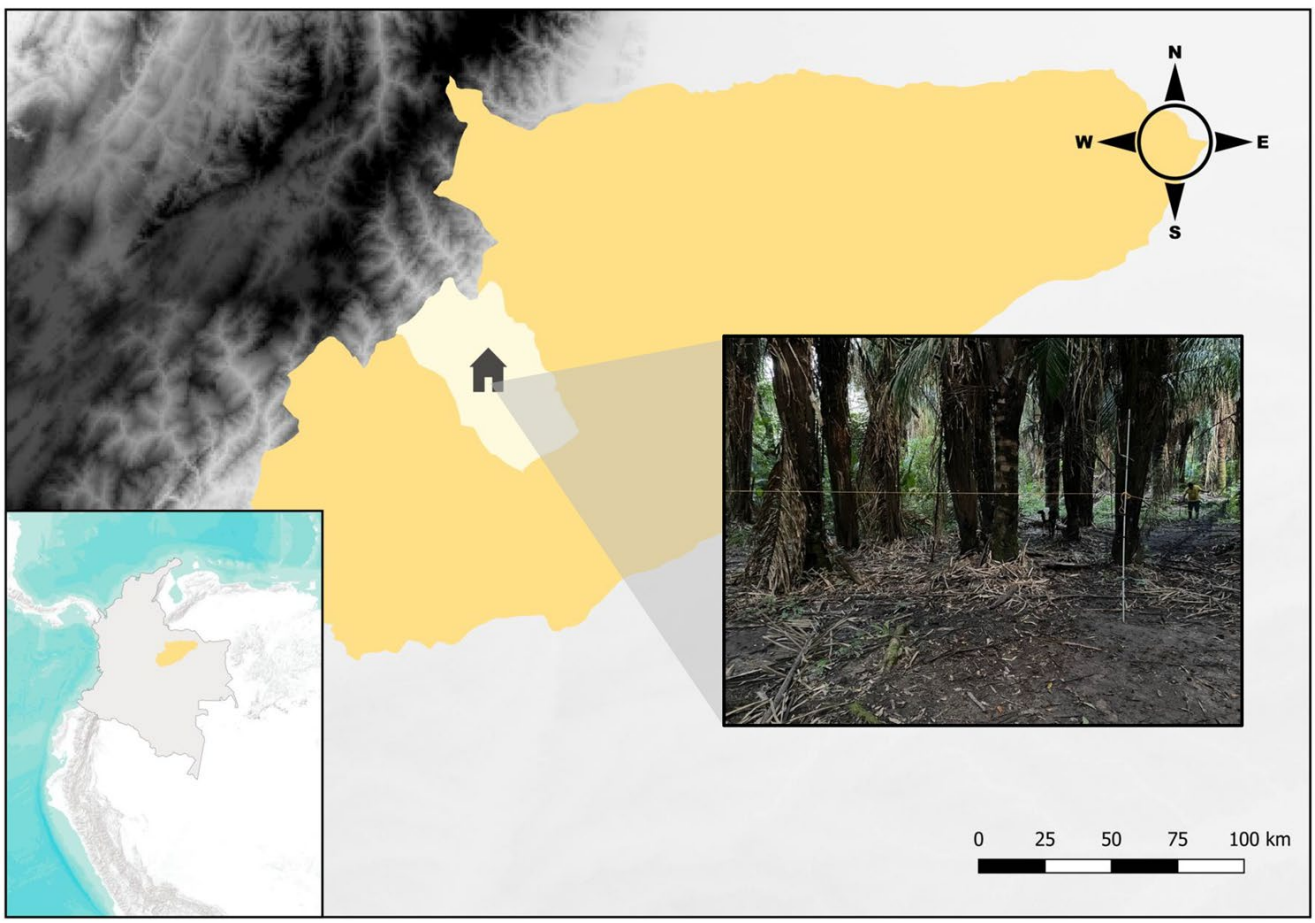
Geographical distribution of the 27 hematophagous bats collected in the rural property “La Cienaga” in the department of Casanare, Colombia. The image illustrates the sampling site

The analysis of microbial communities in reservoirs and/or hosts of infectious diseases contributes to identifying potentially pathogenic microorganisms and understanding their ecological features. Understanding the composition and ecological characteristics of these communities in hematophagous bats not only provides insights into microbial community dynamics but also helps elucidate potential transmission routes associated with the feeding habits of *D. rotundus*. Thus, it provides valuable information for the surveillance and mitigation of infectious diseases, especially in endemic areas. In this study, given the lack of information in Colombia, we characterised the microbial diversity and ecological patterns in blood, oral swab, and faeces samples from *D. rotundus* bats, along with their dietary sources, in a rural property located in Casanare, eastern Colombia (Fig. 1).

## Results

Between July and October 2023, we captured 27 individuals across the secondary forest (tropical rainforest) areas of a rural property dedicated to livestock farming, located in Yopal, in the department of Casanare, Colombia (Fig. 1 and Table S1). For each individual, we collected three different sample types: blood, oral swabs, and faeces. In total, 81 samples were obtained (27 blood samples, 27 oral swabs, and 27 faeces).

### Analysis of sequencing data

High-throughput sequencing of 81 bat samples generated an average of 4,215 raw reads per sample for amplicon-based sequencing (16S- and 18S-rRNA), and 78,648 raw reads per sample for viral metagenomics. After filtering, we identified 91 unique prokaryotic sequences (91% of reads classified), 490 unique eukaryotic sequences (10% of reads classified), and 501 unique viral sequences (10% of reads classified).

### Microbial communities profiling and description

**Fig. 2:**
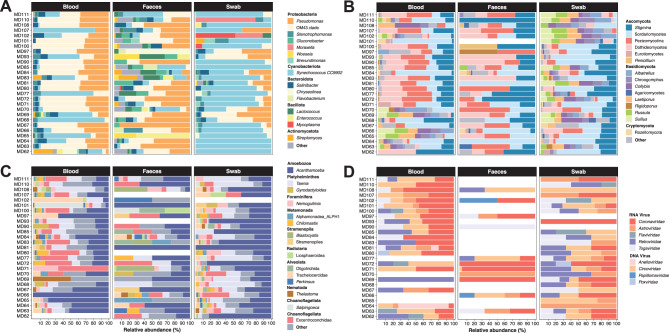
Composition of blood, faeces, and swab microbial communities in *D. rotundus*. Relative abundances of the most abundant (**A**) bacterial genera, (**B**) fungi genera, (**C**) eukaryotic parasites taxa and (**D**) bat viral families across all samples. For each panel, the stacked bar represents the microbial composition of an individual bat. White bars indicate no data

**Fig. 3:**
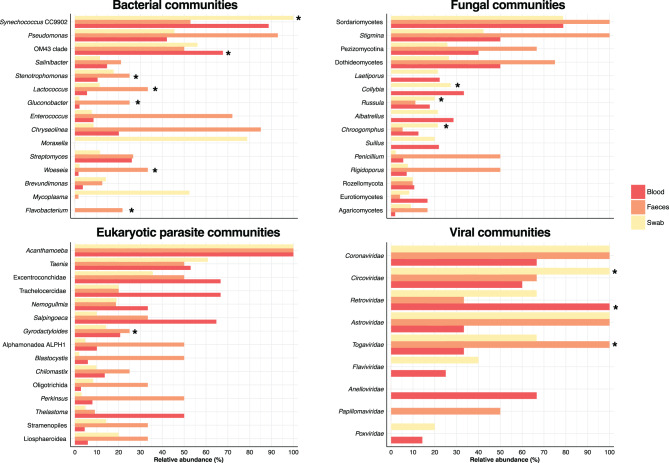
Relative abundance of the 15 most abundant microbial taxa in faeces, blood, and swab samples. Each bar represents the cumulative relative abundance for each taxon across sample types

The full-length 16S-rRNA sequencing showed that Proteobacteria was the most abundant phylum in blood and faeces samples (~64.63% of relative abundance). Bacteroidota (~9.50%) and Cyanobacteriota (~2.56%) followed in abundance, with Cyanobacteriota being the most abundant in swab samples (~25%) (Fig. 2A). At a finer taxonomic scale, the most abundant bacterial genera across the three sample types, with a relative abundance exceeding 60%, were *Bacillus* and *Bifidobacterium*, found mainly in faeces and swab samples, respectively (Figure S1). To further characterize the composition of less represented bacterial genera, we filtered out reads corresponding to these highly abundant taxa. Among the remaining communities, *Pseudomonas*, *Synechococcus* CC9902, and the OM43 clade were the most abundant (Fig. 2A). Statistical analyses revealed significant differences in the relative abundances of these bacterial communities among sample types (Kruskal–Wallis test, *p* < 0.05) (Table S4). Specifically, *Woeseia*, *Lactococcus*, *Stenotrophomonas*, *Gluconobacter*, and *Flavobacterium* were more abundant in faeces samples, while *Synechococcus* CC9902 was more abundant in oral swabs (Fig. 3). In blood samples, only the OM43 clade exhibited a significantly higher relative abundance compared to swab and faeces samples (Fig. 3).

Using full-length 18S-rRNA sequencing, we identified and analysed fungal and eukaryotic parasite (protozoa and helminths) communities across three sample types (Figs. 2B and 2C). Among fungi, Ascomycota (~75.67%) and Basidiomycota (~22.43%) were the most abundant phyla in all samples. For eukaryotic parasites, Amoebozoa (~33%) and Platyhelminthes (~10%) showed the highest relative abundance in blood, faeces, and swab samples. At the genus level, the most abundant fungi in all sample types were *Stigmina*, *Sordariomycetes*, and *Pezizomycetes* (Fig. 2B). In the case of eukaryotic parasites, we found a high number of reads belonging to *Acanthamoeba* and *Taenia* across the three sample types (Fig. 2C). Statistical analyses indicated significant differences in the relative abundances of these microbial communities among sample types (Kruskal–Wallis test, *p* < 0.05) (Table S4). Specifically, *Gyrodactyloides* was more abundant in faeces samples, whereas *Chroogomphus*, *Collybia*, and *Russula* were more abundant in oral swabs (Fig. 3). No significant differences in the relative abundance of fungi and eukaryotic parasites were observed in blood samples (Fig. 3).

In viral communities, we identified a range of viral families across blood, faeces, and swab samples (Fig. 2Dand Figure S2). These communities were mainly composed of RNA viruses (~55% relative abundance), including *Coronaviridae*, *Retroviridae*, and *Togaviridae*. Likewise, we found DNA viruses (~45% relative abundance), with the most abundant families being *Anelloviridae*, *Circoviridae*, *Orthoherpesviridae*, and *Poxviridae* (Fig. 2D and Figure S2). Statistical analyses revealed significant differences in the relative abundances of these viruses among sample types, with *Retroviridae* being significantly more abundant in blood, *Circoviridae* in swabs, and *Togaviridae* in faeces (*p* < 0.05, Kruskal-Wallis) (Table S4 and Fig. 3).

### Changes in the diversity metrics of microbial communities

**Fig. 4:**
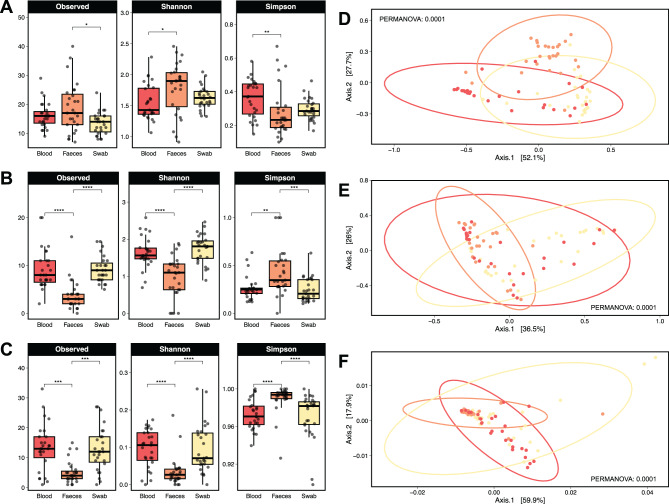
Diversity metrics across blood, faeces, and swab samples of *D. rotundus*. Alpha diversity indices: number of taxa (observed), taxonomic diversity (shannon-wiener), and taxonomic dominance (Simpson) for (**A**) bacteria, (**B**) fungi, (**C**) and eukaryotic parasites. Principal coordinate analysis (PCoA) based on dissimilarity (bray-curtis distances) for (**D**) bacteria, (**E**) fungi, (**F**) and eukaryotic parasites. Significance codes: “*” *p* < 0.05; “**” *p* < 0.01; “***” *p* < 0.001

The diversity metrics showed a higher diversity of bacteria in comparison with the diversity of eukaryotic (fungi and parasites) across the three sample types (Figs. 4A, 4B and 4C). The mean values for the Shannon and Simpson indexes indicate that, in general, the diversity of bacteria, fungi and eukaryotic parasite communities was low in the three sample types (Table S2). Statistical analyses indicated significant differences in the number of taxa, diversity and dominance metrics of bacteria and eukaryotic communities across sample types (*p* < 0.05, Kruskal-Wallis) (Table S2). Specifically, we found differences in assessing the alpha diversity indexes in faeces in relation with blood and swab samples (Figs. 4A, 4B and 4C). On the other hand, principal coordinate analysis (PCoA) with bray-Curtis distances showed differential clustering associated with the sample type in bacteria communities (PERMANOVA, R^2^ = 0.22796, F = 11.515, *p* < 0.0001) (Fig. 4C). Likewise, we found differential clustering in fungi (PERMANOVA, R^2^ = 0.16514, F = 7.7146, *p* < 0.0001) and parasite (PERMANOVA, R^2^ = 0.16514, F = 7.8083, *p* < 0.0001) communities associated with sample type (Figs. 4D and 4F). In particular, we found differential clusters associated with faeces-swab and faeces-blood, respectively (Table S3).

### Identification of pathogenic microbes

**Fig. 5:**
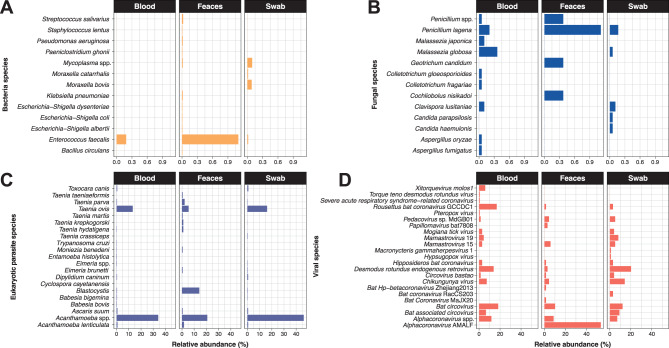
Relative abundances of microbial species pathogenic to mammals. Microbial pathogens in (**A**) bacteria, (**B**) fungal, (**C**) eukaryotic parasites, and (**D**) viral communities across all bat samples

From the composition of microbial communities, we identified various microorganisms considered pathogens to humans and other animals. The taxonomic classification of these pathogens revealed different genera and species whose abundances varied by sample type (Fig. 5). Among bacteria, *Enterococcus faecalis*, *Moraxella bovis*, and *Mycoplasma* spp. were the most abundant pathogens in faeces and swab, respectively (Fig. 5A). In terms of fungi, *Penicillium lagena*, *Geotrichum candidum*, and *Cochliobolus nisikadoi* were abundant in faeces; *Clavispora lusitaniae*, *Malassezia globosa*, and *Malassezia japonica* in blood; and *Clavispora lusitaniae* and *Penicillium lagena* in swab (Fig. 5B). Similarly, in eukaryotic parasite communities, we identified various pathogenic species (Fig. 5C). Among them, *Acanthamoeba* spp. were the most abundant across all sample types ( > 20%). Additionally, we also found other abundant eukaryotic parasites such as *Blastocystis*, *Acanthamoeba lenticulate*, and *Taenia ovis*, with relative abundances above 1% in faeces, blood and swab samples (Fig. 5C). Finally, we also found several pathogenic viruses (Fig. 5D), with *Alphacoronavirus* AMALF, *Bat circovirus*, *Desmodus rotundus endogenous retrovirus*, and *Rousettus bat coronavirus* GCCDC1 being the most abundant ( > 15% of relative abundance).

**Fig. 6:**
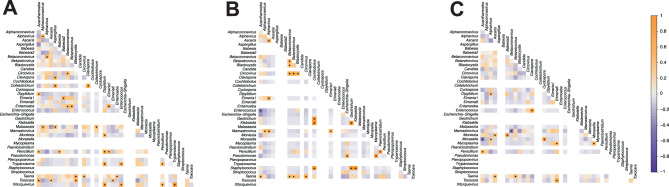
Correlation analysis of the abundances of microbes reported as pathogens of mammals. Each matrix corresponds to the correlations between pathogen abundance in blood (**A**), swab (**B**), and faeces (**C**) samples. Significance code: “*” *p* < 0.05

To evaluate potential interactions among these pathogens, we performed a correlation analysis based on the relative abundance of these communities. Across the three sample types, we found various inter- and intra-domain correlations (Fig. 6). Most significant correlations were positive (*p* < 0.05, *R*_s_ > 0.4). In blood microbial communities, we found inter -domain correlations including three to four taxa, mainly among different eukaryotic parasites and viruses (Fig. 6A). As for faeces, most interactions were inter-domain (e.g., bacteria–eukaryotic parasite, bacteria–fungi, and virus–eukaryotic parasite) than intra-domain (e.g., virus–virus and bacteria–bacteria) (Fig. 6B). For instance, *Betacoronavirus, Betaretrovirus,* and *Circovirus* showed positive correlations with *Taenia*. Additionally, we observed a significant negative correlation (*R*_*s*_ < −0.4) between *Enterococcus* and *Acanthamoeba*. Finally, in swab (Fig. 6C), we found few significant correlations, mainly between eukaryotic parasites, bacteria, and viruses. For instance, *Moraxella* exhibited positive correlations with *Alphavirus* and *Cyclospora* but showed a negative correlation with *Acanthamoeba*.

### Analysis of the feeding sources of *D. rotundus*

**Fig. 7:**
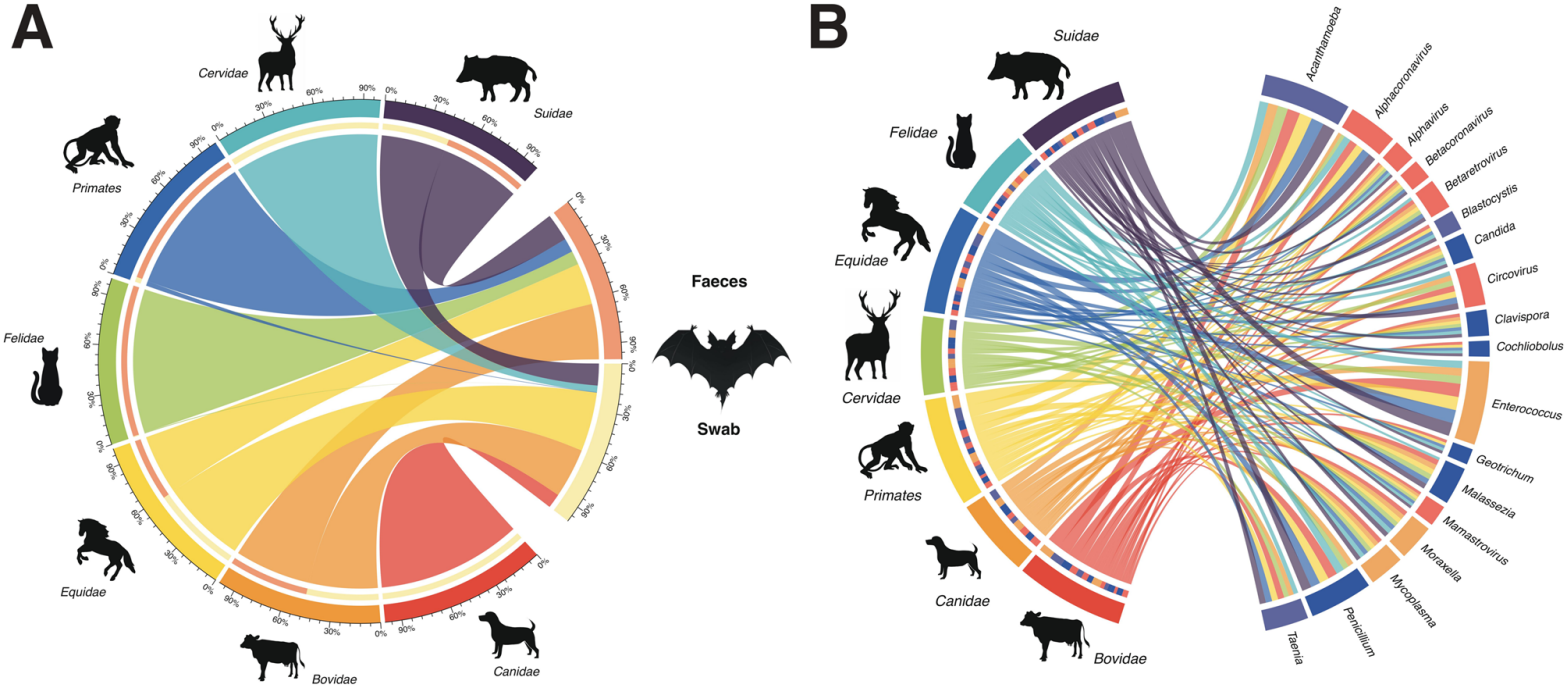
Feeding sources and pathogen associations in *Desmodus rotundus*. Circos plot showing the composition of feeding sources in swab and faeces samples of *Desmodus rotundus* (**A**) and representing associations between vertebrate feeding sources (as potential hosts) and pathogenic microbial genera (**B**). Only microbial taxa with relative abundance > 10% and present in more than one host were included. Colours indicate broad microbial groups: bacteria (orange), fungi (blue), parasites (purple), and viruses (red). The width of the connecting ribbons represents the relative abundance of each microorganism associated with each host taxon

Using 18S taxonomic assignments from faeces and swab samples, we identified feeding sources of *Desmodus rotundus*. The composition of these sources showed a consistent pattern across both sample types (Fig. 7A), with a clear preference for mammals. Among them, Bovidae (~34.5%), Equidae (~33%), and Suidae (~16.3%) were the most frequent. When comparing sample types, swab samples exhibited a higher diversity of feeding sources compared to faeces samples, which included only five distinct sources. As for the relationship with pathogens, the analysis showed multiple networks between the most abundant pathogens ( > 10%) and the different mammalian hosts (Fig. 7B). However, no specific associations were found between pathogens (bacteria, fungi, viruses, and eukaryotic parasites) and food sources of *D. rotundus*.

## Discussion

The common vampire bat exhibits a unique evolutionary adaptation that has driven changes across multiple biological levels, ranging from its genome to its ecological interactions [13]. These adaptations have enabled these bats to harbour a wide range of microorganisms, particularly pathogens, thereby associating them as a reservoir for numerous infectious agents. This has prompted the analysis of the ecological, genomic, and evolutionary aspects of the microbial communities associated with bats. Here, we characterised the microbial communities (bacteria, fungi, eukaryotic parasites, and viruses) in faeces, swab, and blood samples of *Desmodus rotundus*, along with their dietary patterns, collected in an endemic area of infectious diseases. Our analyses highlight four main findings: i) the ecological characteristics of microbial communities, specifically their taxonomic composition and diversity profiles; ii) the presence of several pathogens of public health relevance; iii) patterns of correlations among pathogens, which may suggest potential ecological associations; and iv) potential routes of pathogen transmission.

The bat microbiome comprises of diverse microbial communities, primarily consisting of transient opportunistic taxa or microbes that are pathogenic to other mammals [9, 50, 51]. The taxonomic profile observed in this study, including Proteobacteria, Ascomycota, RNA viruses, and various eukaryotic parasites, suggests the coexistence of multiple microbial assemblages within the common vampire bat. This pattern is consistent with the general composition reported for bat-associated microbial communities [9, 10, 52]. However it varies at finer taxonomic levels, which may reflect ecological and evolutionary specialisations among bats, particularly those linked to dietary strategies [45, 46]. The adaptation to hematophagy has also shaped the microbiome through genomic modifications in metabolic pathways that favour microorganisms adapted to low-carbohydrate and protein-rich environments [13]. In our study, relative abundances of microbial communities in faeces and swab samples indicate specialised functions that are critical to digestion and detoxification of blood components. In faeces, *Enterococcus* and *Gluconobacter* are associated with protein processing and the synthesis of essential compounds [53, 54], while in swabs, *Pseudomonas*, *Stenotrophomonas*, and *Penicillium* may indicate microbial communities capable of forming biofilms and breaking down residual blood components [55–58]. Furthermore, blood consumption also entails high exposure to blood-borne pathogens. These bats have evolved unique immune traits that enable them to regulate and tolerate a range of microorganisms [13, 59], thereby supporting the persistence of potentially pathogenic species. Collectively, these findings highlight that the microbial communities of *D. rotundus* not only provide nutritional compensation for its extreme diet but also reflect its role as an ecologically specialised host, maintaining functionally distinct communities across multiple body niches.

In addition to their functional roles, the ecological patterns of microbial diversity also reflect the influence of dietary specialisation and host physiology. At the ecological level, relative abundances and diversity metrics (alpha and beta) revealed differences in microbial diversity and composition depending on the type of sample analysed (Figs. 2 and 4). Similar patterns in bat microbial ecology suggest that nutrient availability, immune activity and enzymatic processes influence microbial ecology [47, 49]. In our study, faeces samples showed significant differentiation, which may be related to features of the gastrointestinal system and the composition of prey blood. These traits facilitate nutrient breakdown and absorption while shaping microbial community structure [13, 46]. Blood, rich in proteins and iron, generates specific intestinal conditions favouring microorganisms able to metabolise such compounds [13, 45]. Overall, these results indicate that the ecological patterns found in faeces samples may be influenced by the interaction between a specialised diet and the physiological properties of the digestive tract. In contrast, microbial communities found in the other sample types may be more strongly influenced by external factors, such as environmental exposure or the activity of secretory glands.

According to the ecological characteristics of the microbial communities, we identified several taxonomic groups of public health relevance (Figs. 2 and 5. Among them were bacterial species such as *Enterococcus faecalis*, *Escherichia–Shigella coli*, and *Klebsiella pneumoniae*, which have been previously characterised in bats [60], identifying them as potential reservoirs and dispersers of antimicrobial-resistant strains [61]. Although our analyses did not directly assess the presence of resistance genes in *D. rotundus*, the detection of these taxa highlights their potential role in the circulation of such bacteria. Furthermore, we found opportunistic fungi, such as *Candida parapsilosis* and *Clavispora lusitaniae*, which are known to cause infections in humans and have likewise been reported in bats [62, 63]. In addition, we identified several parasitic eukaryotes associated with infectious diseases in both animals and humans [9, 64–66], such as *Trypanosoma cruzi*, *Taenia ovis*, *Acanthamoeba* spp., *Babesia bovis*, and *Blastocystis*. The relative abundances of these pathogens across sample types not only indicate their presence and circulation in the ecosystem but also reinforce the role of bats as hosts or reservoirs of these microbes. Similarly, we identified several viruses of medical relevance, including *Alphacoronavirus* AMALF, *Bat circovirus*, and *Rousettus bat coronavirus* GCCDC1, all widely documented in various bat species, further supporting their ecological role in transmission cycles [39, 52]. These findings highlight not only the diversity of pathogenic microorganisms in *D. rotundus* but also distinct abundance patterns across sample types, suggesting specific ecological dynamics.

The characterisation of the dietary sources of *D. rotundus* provides insights into the potential transmission of pathogens to other organisms. Based on the analysis of swab and faeces samples (Fig. 7), we identified several mammalian families that constitute its feeding habits, mainly domestic mammals and livestock. As a result of intensive cattle farming and deforestation in their habitats, as observed in the department of Casanare, these bats have altered their diet towards livestock consumption [67], which has had an impact on rural economies. Previous studies have shown that ecosystem disturbance not only alters bat distribution but also modifies their food sources [21, 67]. In addition, considering the diversity of pathogens found in these individuals and their lack of host specificity, their feeding behaviour suggests the possible transmission of infectious agents through saliva, faeces, or vector activity, particularly those associated with livestock diseases, such as *Alphacoronavirus* [68], *Circovirus* [69], *Mycoplasma* spp. [70], *Moraxella bovis* [71], and parasitic eukaryotes such as *Babesia bovis* and *Taenia ovis* [72, 73]. Consequently, it is probable that disease circulation may occur among livestock, with bats potentially acting as key transmitters and dispersers of these agents, especially in endemic regions. Furthermore, the loss of natural ecosystems, combined with the ability of these bats to adapt to different environments, increases the probability of transmission to other organisms, compromising their health through the spread of zoonotic microbes. These findings highlight the importance of further understanding of both the ecological and biological characteristics of bats and of the pathogens they carry, which is essential for developing prevention and mitigation strategies against bat-borne infectious diseases.

The different abundance patterns identified for each pathogen, which may be generalist or specific, suggest possible mechanisms of transmission or dispersion, as well as the coexistence of multiple pathogenic microbes. Notably, variation in relative abundance indicates that different sample types may serve as potential transmission routes [47], contributing to the spread of these microbes within ecosystems or to other organisms. These differences may also point to pathogen-specific transmission dynamics. For instance, we identified microorganisms associated with specific sample types and transmission routes: those present in faeces, such as *Blastocystis* are typically transmitted via the faecal–oral route [66, 74, 75]; microorganisms in swab samples, including *Mycoplasma* spp., may be transmitted through biting or licking [76]; and those found in blood, such as *Trypanosoma cruzi* and piroplasmids, are transmitted by arthropod vectors [27, 28]. In contrast, we also found generalist pathogens, mainly viruses and bacteria, capable of using different physiological systems as a transmission routes, suggesting greater complexity in their dispersal and infection mechanisms. Considering the ecological traits of *D. rotundus*, the frequency of these pathogens implies their potential circulation within ecosystems where these bats forage, likely facilitated by faecal–oral transmission or the activity of associated vectors. These findings reveal the variety of infection and transmission routes from bats to other hosts, which could contribute to the emergence of disease outbreaks.

Moreover, our analyses revealed the presence of a broad range of pathogenic species, including bacteria (*Enterococcus faecalis*, *Escherichia–Shigella coli*, and *Klebsiella pneumoniae*), viruses (*Alphacoronavirus* AMALF, *Bat circovirus*, and *Rousettus bat coronavirus* GCCDC1), fungi (*Candida parapsilosis* and *Clavispora lusitaniae*), and parasites (*Acanthamoeba* spp. and *Blastocystis*), within single samples which suggests potential ecological and evolutionary interactions among these microbes [47, 77]. This pattern not only indicates possible transmission routes but also highlights the taxonomic diversity of pathogens in these mammals [78]. Furthermore, such co-existence may involve molecular interactions that influence the persistence, virulence, and transmission capacity of each species. These processes could promote events such as recombination or horizontal gene transfer, particularly among RNA viruses and bacteria [79, 80], which facilitate the emergence of associated diseases. From an eco-epidemiological perspective, the potential transmission routes and the coexistence of pathogens identified in this study underscore the role of these bats as potential hosts or reservoirs of infectious agents, carrying important implications for public health and the emergence of zoonotic diseases. As such, it is essential to incorporate genomic approaches, together with molecular, ecological, and demographic tools, to thoroughly assess the genomic and evolutionary aspects of these pathogens and elucidate the mechanisms governing their ecology.

In the context of microbial coexistence, correlation analyses revealed multiple potential interactions among the pathogenic microorganisms identified in the samples (Fig. 6). These patterns likely reflect ecological processes such as competition, mutualism, or antagonism [81, 82], which can influence the abundance and distribution of these microbes in hematophagous bats. Furthermore, these interactions may also affect pathogen persistence and transmission dynamics, thereby providing eco-epidemiological insights into the mechanisms that underlie coexistence in these hosts. Nevertheless, further functional validation is required to establish the biological significance of these correlations.

The diversity of pathogenic microbes identified across the various samples highlights the immunological capacity of bats to tolerate or control multiple infections. In general, these mammals possess both genomic and cellular mechanisms that enable them to modulate immune responses and maintain a balance between tolerance and resistance to pathogens [11], thereby preventing excessive inflammatory reactions [83]. Previous studies have shown that bats exhibit unique adaptations, including the differential regulation of inflammation-related genes, constitutive expression of interferons, and the expansion of gene families involved in molecular pattern recognition, such as Toll-like receptors (TLRs) and NOD-like receptors (NLRs) [84], along with antiviral genes such as *APOBEC3* and *PKR* [39, 85, 86]. These immunological traits may explain their capacity to harbour a wide range of pathogens without developing clinical symptoms, which in turn influences their ecological role as reservoirs. In *D. rotundus*, these mechanisms may be particularly well-adapted to its hematophagous diet and its frequent exposure to a variety of pathogens in the blood of its prey. This would allow them to coexist with multiple infectious species without compromising their physiological integrity, health status, or fitness [12, 13, 59].

Finally, our findings reinforce the importance of *Desmodus rotundus* as a potentially ecologically specialised host and reservoir, whose physiological and behavioural adaptations influence the structure, diversity, and functionality of its microbial communities. The identification of a diversity of microorganisms, including pathogens of public health interest, as well as evidence of potential transmission routes and microbial coexistence, highlights the role of this bat in the dynamics of infectious diseases.

This study has some limitations that should be considered: (i) although individual-level patterns in microbial communities were identified, the limited number of bats and populations analysed prevented associations with broader ecological variables, such as geography or social structure, which may influence the microbial ecology of these mammals [87]; (ii) no information was available on the frequency and recurrence of infectious outbreaks or on the health status of individuals, which could have clarified the patterns identified in this study; (iii) the biological activity and infective potential of the identified agents remain unknown, which are key aspects for determining their virulence, transmission mechanisms and the epidemiological role of these bats; and iv) although different viruses were identified using enrichment techniques and reference databases, some groups such as bacteriophages were not examined in detail, and potential biases together with the absence of complete viral genomes limit deeper ecological and evolutionary interpretation. Despite these limitations, this is the first study to describe the ecological characteristics of microbial communities (bacteria, fungi, eukaryotic parasites, and viruses) present in the blood, faeces, and oral secretions of *D. rotundus* in endemic areas for infectious diseases, together with its feeding sources and the potential pathogens it harbours. Overall, these findings underscore the need to continue investigating the microbial communities of these and other reservoir mammals, in order to further our understanding of their ecology and biology.

## Conclusion

Microbial communities in *Desmodus rotundus*, across blood, faeces, and swab samples, are composed of diverse bacteria, fungi, protozoa, and viruses, including several pathogenic taxa. The abundance, diversity, and potential interactions of these communities varied among sample types, suggesting an influence of physiological factors and indicating possible routes of microbial transmission. Moreover, the coexistence of multiple pathogens within individual bats, together with correlations between taxa, underscores the complexity of microbial interactions in this species. Finally, the identification of livestock as primary feeding sources may indicate possible transmission and circulation of pathogenic microorganisms, particularly those associated with livestock diseases. This study has certain limitations, including a small sample size, the lack of comparative populations, and the absence of functional or epidemiological data. Future research incorporating hologenomic approaches and population ecology is needed to understand the ecological, genomic, and evolutionary dynamics of these communities and to clarify the role of *D. rotundus* in pathogen transmission cycles.

## Methods

### Capture and sampling of bats

A total of 27 common vampire bats (*Desmodus rotundus*) were captured using mist nets placed in forested areas of the rural property “La Ciénaga” (5°14’59.46’‘N, 72°17’1.781’‘W), located in Yopal in the department of Casanare, Colombia, between June and October 2023 (Fig. 1 and Table S1). The property comprises fragments of secondary forest (tropical rainforests) and areas dedicated to livestock farming, mainly cattle, under intensive grazing practices. Following capture, the bats were anaesthetised with ketamine. Subsequently, the following procedures were performed on each individual: oral secretion collection via oropharyngeal swabbing, blood extraction via cardiac puncture using an insulin syringe (~500 µL), and faeces sampling directly from each bat during handling. After sample collection, all individuals were released at the same sampling site following full recovery from anaesthesia. Capture and sampling procedures were approved by the Ethics Committee (Approval No. DVO005 1585-CV1427). We confirm that the study is reported in accordance with ARRIVE guidelines. With the exception of the faeces samples, all samples were preserved in 1 mL of Zymo Shield solution (DNA/RNA Shield, Zymo Research) to stabilise nucleic acids for downstream analysis.

### Nucleic acid isolation from bat samples

The genetic material from blood, oral swab and faeces samples was isolated using the protocol described by Luna et al., 2024. Briefly, we used the half of the sample volume for DNA extraction and the remaining half for RNA extraction. For DNA extraction, the High Pure PCR Template Preparation kit (Roche Life Science) was employed for blood and swab samples, and the Stool DNA Isolation Kit (Norgen, Biotek Corp.) for faeces samples. For both extraction protocols, we followed the manufacturer’s instructions, with an elution volume modified to 100 µL. In terms of RNA extraction, the Quick-RNA Viral Kit (Zymo Research) was employed with some modifications. Briefly, a preprocessing step was incorporated depending on the type of sample. Faeces samples were incubated in 200 µL of PBS 1X for 12 hours and homogenized by disruption with ceramic beads for 5 minutes at 30 Hz using a TissueLyser II disruptor (Qiagen). Blood and swab samples were mechanically homogenized by pipetting for 30 seconds to release the biological material. Afterward, the homogenized samples were incubated with 5% v/v of proteinase K (20 mg/ml, Zymo Research) at room temperature for 15 minutes. Finally, we followed the manufacturer’s instructions with a final elution step of 20 µL of DNase- and RNase-free water, previously preheated to 56 °C.

The concentration, quantity and integrity of the extracted nucleic acids were assessed using 1.5% agarose gel electrophoresis and NanoDrop One spectrophotometry. To ensure the absence of cross-contamination between samples, we included negative controls with molecular-grade water during the extraction process.

### Long-read amplicon sequencing of 16S-and 18S-rRNA genes

To characterise prokaryotic and eukaryotic communities, we used DNA samples for amplicon-based sequencing with Oxford Nanopore Technologies (ONT). The sequencing process involved PCR amplification of the full-length 16S-rRNA gene (~1.5 kb) and the V4–V5 hypervariable regions of the 18S-rRNA gene (~0.7 kb), using universal primers 27F and 1492 R for 16S [88] and 566F and 1289 R for 18S [89]. For both genes, PCR reactions were performed using LongAmp Taq 2X Master Mix (New England BioLabs), 0.4 µM of each primer, and the template DNA. Amplification conditions consisted of an initial denaturation at 94 °C for 1 minute, followed by 30 cycles of 94 °C for 30 seconds, 54 °C for 1 minute (18S-rRNA) or 48 °C for 1 minute 30 seconds (16S-rRNA), and 65 °C for 1 minute, with a final extension at 65 °C for 10 minutes. Throughout the PCR reaction, a negative control with molecular-grade water was included as an additional precaution against PCR contaminants. Amplicons were visualised on a 2% agarose gel, then purified and prepared for ONT sequencing following the manufacturer’s instructions. First, the NEBNext End Repair/dA-Tailing Module was used to end-repair and A-tail the PCR products. Unique barcodes from the PCR Barcoding Kit (EXP-NBD196) were then added using the NEB Blunt/TA Ligase Master Mix, followed by adapter ligation with the NEBNext Quick Ligation Module Kit (SQK-NBD114.96). The final library was loaded onto a FLO-MIN114 R10.4.1 flow cell on the MinION™ MK1C device (ONT) and sequenced using the MinKNOW v24.02.16 for 72 hours.

Following sequencing, raw signal files (POD5) were processed using Dorado v0.0.7.0 (https://github.com/nanoporetech/dorado) with the Super accurate model for basecalling and demultiplexing. We assessed the quality scores of ONT sequencing data using NanoPack2 v0.1.1.1 [90]. Next, we used emu v0.3.4.5 [91] to perform species-level microbial community profiling. We used the default parameters for taxonomic assignment, aligning high-throughput data against the SILVA database version 138.1 for 16S-rRNA [92, 93] and PR2 database version 5.0.0 for 18S-rRNA [94] using minimap2 v2.22. This software employs an expectation-maximization (EM) algorithm to estimate species abundances, improving accuracy by directly assigning sequences to taxa without clustering [95].

### Enrichment and sequencing of viral communities

RNA samples were used for the enrichment and sequencing of viral communities in bats. All samples were enriched using Rapid-SMART9n, a method that enables the amplification and sequencing of diverse viruses [96]. Briefly, for cDNA synthesis, RNA was mixed with the primer RLB-RT9N (2 µM) and dNTPs (10 mM) (New England BioLabs), incubated at 65 °C for 5 minutes, and then cooled on ice. The annealed RNA was combined with SuperScript IV First-Strand Buffer (5X), DTT (100 mM), RNase OUT, RLB TSO (2 µM), and SuperScript IV RT (200 U/µL) (Invitrogen, Carlsbad), and incubated at 42 °C for 90 minutes followed by 10 minutes at 70 °C to synthesise the cDNA. The resulting cDNA was then amplified using LongAmp Taq 2X Master Mix (New England BioLabs), Nuclease-free water (NFW), RLB primer (10 µM), and the synthesised cDNA. PCR cycling conditions were as follows: initial denaturation at 98 °C for 45 seconds; 30 cycles of 98 °C for 15 seconds, 62 °C for 15 seconds, and 65 °C for 5 minutes; and a final extension at 65 °C for 10 minutes. During the enriched process, all samples were quantified using the Qubit dsDNA High Sensitivity Assay (Life Technologies) on the Qubit 3.0 fluorometer to ensure proper execution.

The enrichment samples were used for sequencing by ONT. We prepared ONT libraries following the manufacturer’s instructions. Initially, the DNA ends were prepared using the NEBNext® Ultra™ II End Repair/dA-Tailing Module commercial kit. Then, a unique barcode from the Native Barcoding Kit (EXP-NBD104) was added for each sample using the NEBNext® Ultra™ II Ligation Module kit. Finally, adapters were ligated using the NEBNext® Quick Ligation Module kit in conjunction with the ligation kit (SQK- LSK109). The libraries then were loaded into FLO-MIN106 flow cells R9.4.1 on the MinION™ MK1C device (ONT) and sequenced using MinKNOW V0.3.1.4. program for 72 hours.

After sequencing, raw signal files (POD5) were processed using Dorado with the Super accurate model for basecalling and demultiplexing. Next, we assessed the quality scores of ONT sequencing data using NanoPack2 [90]. Reads aligned to the reference genome from the GenBank assembly, *Desmodus rotundus*: GCF_022682495.2, were filtered out using Minimap2 v0.2.24 [97] and SAMtools v0.1.21 [98]. An additional filtering step was performed with SortMeRNA v0.4.3.7 [99] to eliminate residual prokaryotic and eukaryotic ribosomal RNA reads. To analyse the viral communities, a taxonomic assignment was carried out from the filtered reads using emu [91]. Two custom databases were utilized for taxonomic assignment: the Bat virus database and the Refseq virus database. The Bat virus database contained 1,026 dereplicated and complete viral genomes or segments reported in Chiroptera, retrieved from NCBI Virus as of July 9, 2024 (https://github.com/gimur/Desmodus_Dataset_2025/tree/main/Bat_DB). The RefSeq virus database included 17,553 dereplicated and complete viral genomes or segments from RefSeq–NCBI Virus as of the same date (https://github.com/gimur/Desmodus_Dataset_2025/tree/main/RefSeq_DB).

### Description of microbial communities’ features

In order to describe the microbial community features, we first filtered out the taxa corresponding to mitochondria, chloroplast, algae, plants, and dinoflagellates from the abundance and taxonomic assignment tables using the R *phyloseq* package v0.1.40.0 [100]. To characterise the composition of microbial communities, we identified the 15 most abundant genera in blood, swab, and faeces samples from each individual. This identification was based on the proportion of reads of each taxon (relative abundance) to the total sample dataset. Likewise, we evaluated the differences in the abundances of each taxonomic group using the non-parametric test Kruskal-Wallis with Dunn test as post hoc with Benjamini–Hochberg (fdr) correction. To estimate the alpha (α) diversity metrics, we used the Observed (number of taxa), Shannon-Wiener (species diversity) and Simpson (species dominance) indices from the *microbiome* package of R v0.1.18.0. Differences between sample types and ecological area were assessed using the same non-parametric test. In terms of beta (β) diversity, the dissimilarities of the microbiota were assessed and visualized by principal coordinate analysis (PCoA) of the *phyloseq* package. Furthermore, we applied a permutational multivariate analysis of variance test (PERMANOVA), from the *vegan* package v0.2.6–2 [101], with 9,999 permutations to assess changes in microbiota communities according to sample type and ecological area. Both PCoA and PERMANOVA analyses were performed on Bray-Curtis distances obtained from the relative abundances of each taxon.

### Identification and interaction of pathogens microbes

Based on the composition of the microbial communities, we examined the taxa previously reported in the literature as mammalian pathogens detected in bats (bacteria, fungi, protozoa and viruses) [11, 102]. Reads associated with these taxa were then re-classified with EMU (default settings) using a RefSeq-derived reference database [103] to ensure consistent genus- or species-level assignments. Pathogen selection relied exclusively on the literature; no virulence or functional assessments were performed. The taxonomic assignments and abundance values were examined in RStudio v0.4.4.2. To explore potential ecological associations among these taxa, we calculated Spearman’s rank correlations using read counts and visualised the results with the *corrplot* v0.0.95 package [104].

### Characterisation of feeding sources

To understand the potential transmission of pathogens to other animals, we analysed the different feeding sources of common vampire bats. Specifically, we used the 18S taxonomic assignments from swab and faeces samples to identify and filter the vertebrate species consumed by *Desmodus rotundus* [17]. The relative abundances of each vertebrate were estimated and visualised using a circos plot from circlize v0.0.4.11 package in RStudio. Furthermore, we assessed potential associations between the identified vertebrates (as potential hosts) and the identified pathogenic microbes. To do this, we integrated the relative abundances of pathogenic microbes with those of the vertebrates identified as food sources. A second circos plot was then constructed to visualise the associations between microbes and vertebrate hosts, considering only pathogens with a relative abundance greater than 10%.

## Electronic supplementary material

Below is the link to the electronic supplementary material.

Figure S1. Composition of bacterial communities in blood, faeces, and swab microbial communities of hematophagous bats. For each panel, the stacked bar represents the microbial composition of an individual bat. White bars indicate no data.

Figure S2. Composition of viral families in blood, swab, and faeces samples of hematophagous bats. This families were assigned using sequences and reference genomes from RefSeq. For each panel, the stacked bar represents an individual bat. White bars indicate no data.

Supplementary Material 1

Supplementary Material 2

Supplementary Material 3

Supplementary Material 4

## Data Availability

The raw sequencing data used in this study are available in the European Nucleotide Archive (ENA) under the project accession number PRJEB81318. The data generated and analysed in this study are accessible at https://github.com/gimur/Desmodus_Dataset_2025/tree/main/Microbiome_data.

## Abbreviations

DNA: Deoxyribonucleic acid
NLRs: NOD-like receptors
ONT: Oxford Nanopore Technologies
PCR: Polymerase chain reaction
PCoA: Principal coordinate analysis
PERMANOVA: Permutational multivariate analysis of variance
RABV: Rabies virus
RNA: Ribonucleic acid
TLRs: Toll-like receptors
