# Supplementary figures and images for: Ecological insights into the cross-domain microbiome interactions in the hematophagous bat Desmodus rotundus
Source: Anim Microbiome. 2026 Feb 19;8:22. doi: 10.1186/s42523-025-00504-x (PMC12922381; doi:10.1186/s42523-025-00504-x)

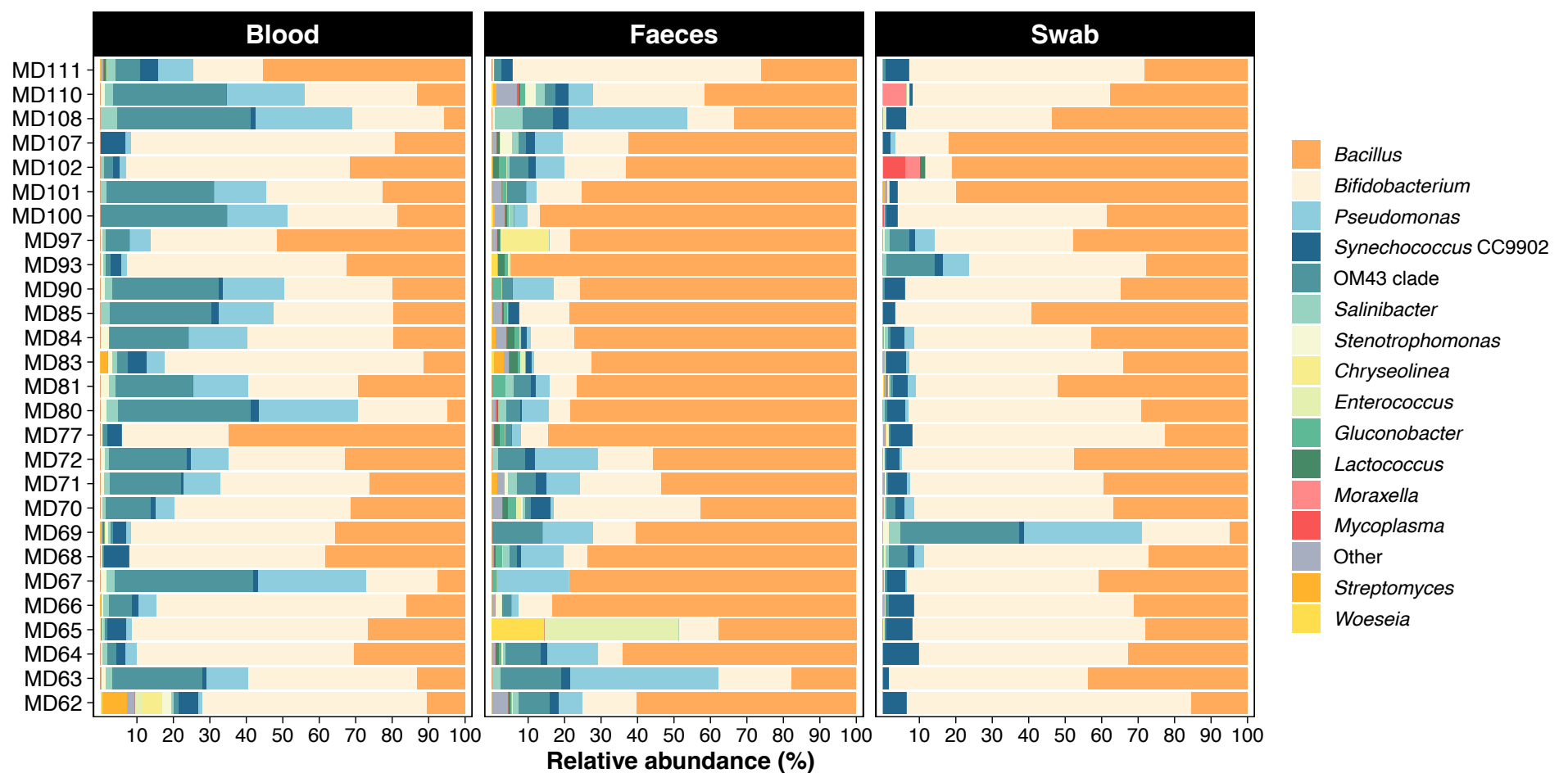

Supplement: Supplementary file 1 — Figure S1. Composition of bacterial communities in blood, faeces, and swab microbial communities of hematophagous bats. For each panel, the stacked bar represents the microbial composition of an individual bat. White bars indicate no data. [file 42523_2025_504_MOESM1_ESM.pdf]

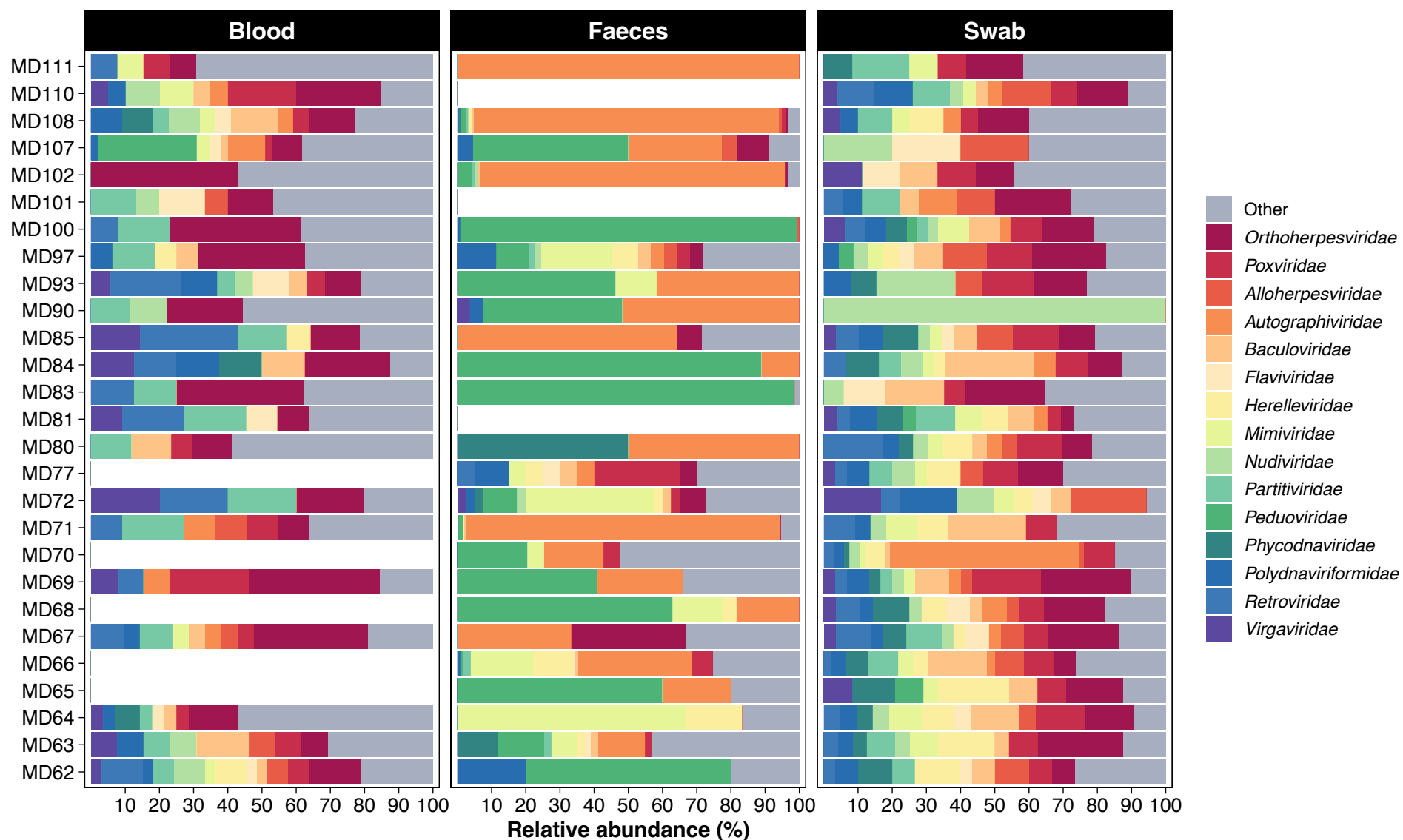

Supplement: Supplementary file 2 — Figure S2. Composition of viral families in blood, swab, and faeces samples of hematophagous bats. This families were assigned using sequences and reference genomes from RefSeq. For each panel, the stacked bar represents an individual bat. White bars indicate no data. [file 42523_2025_504_MOESM2_ESM.pdf]
